# An Evaluation of Natural Environment Interventions for Informal Cancer Caregivers in the Community

**DOI:** 10.3390/ijerph182111124

**Published:** 2021-10-22

**Authors:** Rebecca H. Lehto, Gwen Wyatt, Jessica Sender, Sara E. Miller

**Affiliations:** 1 College of Nursing, Michigan State University, East Lansing, MI 48824, USA; gwyatt@msu.edu (G.W.); jsender@msu.edu (J.S.); 2 Department of Human Development and Family Studies, Pennsylvania State University, State College, PA 16802, USA; sem588@psu.edu

**Keywords:** cancer caregivers, nature, environment, integrative therapies, quality of life, health

## Abstract

Home-based informal caregiving by friends and family members of patients with cancer is be-coming increasingly common globally with rates continuing to rise. Such caregiving is often emo-tionally and cognitively demanding, resulting in mental exhaustion and high perceived burden. Support for caregivers may be fostered by engagement with the natural environment. Interaction with nature is associated with mental health benefits such as stress reduction and improved well-being. The purpose of this paper was to evaluate the state of the science regarding the use of nat-ural environment interventions to support caregivers of cancer patients in the community. A comprehensive scoping review using the Arksey and O’Malley framework and the Preferred Re-porting Items for Systematic Reviews and Meta-analyses assessed natural environment therapies and mental health outcomes among cancer caregivers. Databases searched included CINAHL, PubMed, Scopus, Cochrane, and Alt HealthWatch. Findings recovered a total of five studies over a 10-year period that met criteria, demonstrating a lack of empirical evidence addressing this po-tential resource to support caregivers. Often, study appraisal was not on nature exposure, but ra-ther other aspects of the projects such as program evaluation, exercise, or complementary thera-pies. Both qualitative and quantitative designs were used but sample sizes were small. Caregivers experienced beneficial results across the various studies and future work could enhance these findings.

## 1. Introduction

The natural environment has been recognized to be a source of healing and restoration for centuries [1]. The purpose of this paper was to evaluate the state of the science regarding the use of natural environment interventions to support caregivers of cancer patients in the community. Cancer caregivers who deliver care to patients in the home environment are a growing sector of the global population with unmet needs [2,3,4,5,6,7]. In the United States alone, there are an estimated three million family caregivers caring for cancer patients in the home environment [8]. These caregivers provide a range of unpaid services and are engaged in multifaceted roles that place high demand on cognitive and emotional functioning. Given the continuing trend that has transitioned cancer care into the outpatient arena, demands placed on the home-based caregiver are growing in complexity. Compared with other types of informal caregivers, cancer caregivers are significantly more responsible for performing nursing and medical duties, often with limited or no training [9,10,11]. There is growing awareness of the need for cost-effective strategies to support the cancer caregiver [5,9]. This scoping review of the literature has the potential to guide future research and uncover recommended strategies for integrating natural environment interventions. Such intervention can support informal caregivers in the community who deliver home-based care for patients with cancer.

About 17 million people are diagnosed with cancer yearly, a disease that is the second leading cause of death globally [12]. A diagnosis of cancer is highly distressing and is increasingly viewed as a chronic disease that has well demarcated phases including diagnostic, treatment, survivorship, and end-of-life time points that all carry varying challenges [5,13]. Depending on the type of cancer concomitant with advances in treatment sophistication, patients are receiving increasingly complex treatments. Such treatments may include targeted therapies, immunotherapies, and oral antineoplastic agents that may be initiated in outpatient clinics but with most of the symptom management occurring in the home environment [10,11]. As a disease that profoundly affects families and their community of loved ones, the brunt of supportive care that patients with cancer receive is placed on informal friend and/or family caregivers [7,10,11,14]. Such home-based care includes, but is not limited to, emotional support, communicating with health providers, organizing and/or providing transportation to healthcare appointments, activities of daily living support, medication management, and symptom assessment and management [11]. Younger caregivers are placed in a position to provide this unpaid support while engaging in outside employment, having childcare responsibilities, and other demands on their time and capacity to serve [11]. However, most cancer caregivers are older adults, primarily female, and often have personal health and/or chronic conditions that require maintenance [10].

While the interest in conducting and evaluating research to improve cancer caregiver wellbeing has grown substantively over the past two decades, there remains a priority need for interventions to support emotional health, coping, spiritual needs, stress management, health promotion, and end-of-life [3]. Research evaluating strategies to support caregivers who have less accessible geographic areas, such as natural rural regions that are also cost effective to provide, are priority areas in this regard [3]. Such interventions need to be readily available, flexibly delivered, and capable of assuaging the mental exhaustion and psychological distress associated with the demands of caregiving. Further, such interventions ideally could also be partaken with the cancer patient.

### 1.1. Theoretical Underpinnings

Two complementary theoretical positions that address the restorative benefits of nature have been articulated. One of these theoretical approaches identifies the power of nature to rest and restore one’s directed attention capacity. In this regard, Kaplan and Kaplan refer to the visual-spatial properties of nature as “soft fascinations” that are able to capture involuntary attention and provide repose for voluntary attention demands, a finite resource essential for the complex demands of effective cognitive functioning [15,16]. The second theoretical approach explains mechanisms by which the natural environment has the capacity to reduce perceived stress [17]. Both theories contend that humans are physiologically and psychologically predisposed to pay attention and respond positively to natural environments, characteristic of settings that were favorable to survival during early evolution [15]. Exposure to nature promotes rest, calmness, and relaxation, while enhancing the tendency for reflection, mechanisms by which nature aids beneficial outcomes [18]. Although both theoretical avenues portray natural environments as restorative to human mental health, they are synergistic in that the stress reduction perspective identifies physiological stress, and the attention restoration theory identifies alleviation of mental fatigue as the endpoints of positive outcomes from nature immersion [18]. For example, the heightened physiological arousal and negative affect that are characteristic of stress and mental fatigue both occur in response to emotionally and cognitively taxing caregiving experiences [19], whereas exposure to nature promotes rest, calmness, and relaxation, while enhancing the tendency for reflection, which may be the underlying mechanism by which nature could support constructive mental health outcomes for individuals facing ongoing stressful circumstances such as cancer caregiving.

### 1.2. Background

As with any scoping review, when the literature is scant on the desired topic, nearby literature can shed light on the state of the science. For this cancer caregiver topic, it was necessary to explore around the edges of the precise area of interest. A strong body of evidence revealed the benefits of nature conducted in both indoor and outdoor settings including woodlands [20], water [21], urban green space [22], natural visual scenes [23], and gardens [24,25,26,27]. Moreover, nurse researchers have recommended “nature exposures” as a resource that practicing oncology nurses can use to support cancer caregivers [14,28]. This peripheral and grey literature is useful in a scoping review to reveal not only the state of the science, but also the gaps in the literature related to cancer caregivers, and the potential for nature interventions to ease the burden of cancer caregiving [13,29,30].

## 2. Materials and Methods

A comprehensive scoping review using the Arksey and O’Malley framework [31] was conducted that assessed natural environment intervention to support cancer caregivers. Scoping reviews are useful for conducting preliminary assessment of available research literature for topics that have not been comprehensively reviewed, and/or that are large, complex, and of a heterogeneous nature that is not suitable for systematic reviews. The five stages recommended by Arksey & O’Malley [31] to determine the state of the science around a problem of inquiry include: Stage (1) Identification of the research question and eligibility criteria; Stage (2) Identification of relevant publications; Stage (3) Selection of publications; Stage (4) Charting the data; and Stage (5) Collating, summarizing, and reporting the results.

### 2.1. Stage 1: Identification of the Research Question and Eligibility Criteria

The review was guided by the following research question: What is the state of the science regarding natural environment interventions to support caregivers of cancer patients in the community?

### 2.2. Stage 2: Identification of Relevant Publications

The librarian for the College of Nursing conducted two searches—one on December 3, 2020, and the other on 20 September 2021. The search was conducted in the following databases: CINAHL, PubMed, Alt HealthWatch, Cochrane, and Scopus. There were no date limits on the search. Controlled vocabulary (Medical Subject Headings [MeSH], CINAHL Subject Headings) as well as keywords were used. The search was modified for each database according to controlled vocabulary and search capacities, but remained largely similar across databases.

The searches focused on the following main areas: (1) informal caregivers (lay, family, spouse, etc.); (2) cancer; (3) nature, the environment, ecology, and associated nature keywords; and (4) therapeutic interventions (complementary, alternative, integrative, quality of life). Full keyword searches for each database can be found in the Supplementary (Table S1).

Inclusion criteria focused on: cancer caregivers, nature interventions (indoors or outdoors), and health outcomes. Exclusion criteria included professional caregivers and those that were non-cancer related. The two searches produced 1647 results. This was: CINAHL: 226; Alt HealthWatch: 9; PsycINFO: 293; PubMed: 920; Scopus: 196; Cochrane: 3. After duplicate removal, there were 1275 articles for review. Searching for references through article bibliographies was also conducted, yielding 8 additional references. An additional 773 articles and 8 books were removed due to being off topic or patient rather than caregiver focused, leaving 69 articles. Other reasons for exclusion of articles were non-cancer caregivers, lack of nature experience in the publication, hospital-based programs that did not include the friend or family caregiver, information for caregivers rather than information about caregivers, and studies of attitudes rather than health outcomes.

### 2.3. Stage 3: Selection of Publications

**Figure 1:**
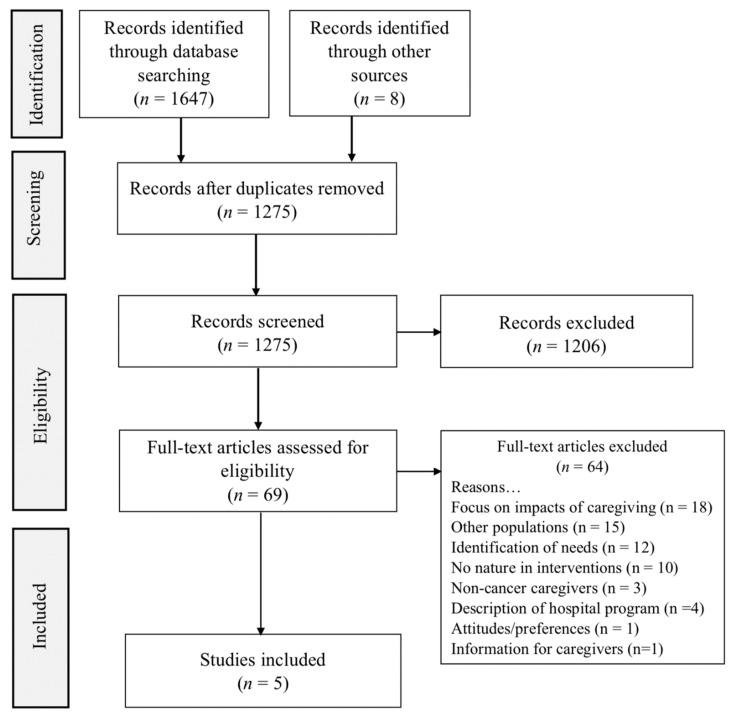
PRISMA-ScR diagram of search strategy.

A modified version of the Preferred Reporting Items for Systematic Reviews and Meta-Analyses (PRISMA) flow diagram—scoping review was utilized to illustrate the selection of publications for the review [32]. The 69 remaining articles were then reviewed for eligibility. Of these 69 papers, 5 were retained for inclusion in the review (see Figure 1).

## 3. Results

### 3.1. Stage 4 and 5: Charting the Data and Collating, Summarizing, and Reporting the Results

Table 1 illustrates the grid used to chart the data and summarize findings. Our review of the literature identified that the research on natural environment therapies was largely concentrated on patient populations rather than informal cancer caregivers. In studies that involved cancer caregivers, one study focused on caregivers of patients at end-of-life [33], two were primarily focused on pediatric patients and their parent caregiver [34,35], one looked at a nature intervention along with other types of complementary therapies [36], and one study involved a physical activity intervention that could include activities in the natural environment [37]. Included sample sizes ranged from 34–106 caregivers who were aged 26–76 years, primarily female, and represented mixed race/ethnicities [33,34,35,36,37]. Four of the five studies on experiences with nature were conducted indoors.

Of the five studies included, the end-of-life study conducted by Lavin and associates in the United States evaluated the effect of group flower arranging on self-efficacy and perceived stress among caregivers of patients who were hospitalized in inpatient hospice care [33]. Utilizing a mixed methods pre-post design, 71 family caregivers participated in a flower arranging program that was provided as part of the complementary modality support services over a 4-month time frame. Participants took the completed flower arrangements that they created to the patients’ hospice room. The flower arrangements were developed in a supportive group, and caregivers were able to share feelings and express emotions while engaged in the activity. Stress levels and problems associated with stress were significantly lower following participation in the intervention. Further, only 16 of the 71 caregivers completed the post self-efficacy survey, so pre-post data were only assessed on the 16 caregivers with complete data on this variable. Findings from the 16 caregivers with pre-post scores demonstrated significant improvement in perceived self-efficacy. Qualitative data revealed that the experience most frequently (*n* = 57) improved mood, engendered a sense of healing, and was life affirming. Caregiver participants identified that the flower arranging reduced worry, was a positive distraction, and was a positive sensory experience for the hospice patient [33].

The first of the two pediatric studies was conducted by McCullough and associates [34] and was a multisite study that evaluated the use of an animal-assisted intervention to support pediatric cancer patients and their parent caregivers [34]. The study utilized a randomized control design where 60 patient/caregiver dyads participated in the intervention and 46 in the usual care control condition. The study occurred in the outpatient clinics where the child received anticancer treatments. Findings demonstrated significant reductions in perceived stress among the parent caregivers including with communications and stressful events associated with medical care [34].

The second pediatric study, carried out in Spain, evaluated the use of a technological play intervention that provided access to observing zoo animals around the world using remote video cameras [35]. Participants included 39 parent caregivers and pediatric oncology patients who were hospitalized on a hematology-oncology unit. Using a mixed methods pre-post design, the parent caregivers and their children who were undergoing cancer treatment engaged in the intervention for a 30-min period in the patient’s hospital room. Outcomes tested included parent-child interactions, anxiety, positive and negative experiences, mood, and depression. Findings demonstrated that the parent caregivers reported positive affect, significant improvements in relating to the child, and high satisfaction from their experiences with the intervention [35].

Another study evaluated the impact of complementary therapy use on quality of life outcomes in cancer caregivers who participated in a hospital program that included outdoor gardens [36]. Using a secondary analysis approach, the study (*n* = 56; 35 complementary therapy users; 21 non-users) identified garden participation as being the most used complementary therapy along with arts programs (40%), demonstrating the popularity of the nature-oriented activity. Participants who participated in the quality of life survey reported better scores on mental function, emotional state, and attitude toward life if they engaged in the hospital complementary therapies program as compared to those who did not [36].

The final study that included outdoor participation was a physical activity study for patients with cancer and their caregivers who received surgery for lung or gastrointestinal cancers [37]. In this study, barriers and facilitators to engagement in the intervention were evaluated based on qualitative interview notes in physical and/or occupational therapy documents. Data were derived from 34 patient-caregiver dyads. Barriers noted to outdoor exercise by caregivers related to physical pain stemming from comorbid conditions and allergy sensitivities. Caregivers noted competing demands from busy schedules as deterrents to participation. Benefits that facilitated outdoor activity included patient motivation to hike and decreases in perceived anxiety [37].

### 3.2. Summary

Findings from the review indicate that very limited research has evaluated restorative natural environment interventions to support cancer caregivers. In fact, only one of the five nature studies included in this review was conducted outdoors. Studies that have included natural environments did so either in the context of a larger focus, such as complementary modalities or exercise, and were not primarily aimed at evaluating outcomes from nature exposure. Further, sample sizes were small, and outcomes were targeted towards the patient and only secondary to the caregiver in three out of the five studies. Strengths of the studies include use of both quantitative and qualitative methodologies to glean information on intervention outcomes. Only one study used a randomized design and two were oriented toward program evaluations. In all studies evaluating cancer caregiver outcomes, positive benefits from the nature interventions were reported.

## 4. Discussion

The purpose of the scoping review was to determine the state of the science regarding the testing of natural environment interventions to support informal cancer caregivers. While a growing body of evidence indicates that natural environments support human health and wellbeing, there is a paucity of information regarding their efficaciousness in supporting informal caregivers of patients with cancer. However, even with these limited studies, it is clear the important role nature may play in the health and well-being of cancer caregivers.

In a healthy population, it has been identified that natural environments have the capacity to support stress management, improve quality of life and mental health, and support cognitive functioning [30]. Caregivers are recognized to experience high stress, which increases accordingly in relation to the burden of caregiving and the need to balance competing demands [10,19].

While only one of the studies in this review involved the outdoors, findings suggest that the outdoor experience was fraught with barriers such as allergies and weather [37]. This finding suggests the need to tailor nature interventions to accommodate those who can most benefit. Study criteria such as allergies and functional status of the caregiver may have to be considered. Further, nature experiences that do not require direct exposure to the weather—such as conservatories or botanical domes—may be useful options especially in harsher climates. Given more than half the global population live in urban regions with more limited options for nature immersion, [22] those caregivers residing in environments with reduced access to greenspace areas may also need personalized support. For example, interventions that involve fieldtrips to local restorative natural settings could be developed and evaluated. Indoor nature projects could be considered with flowers or small trees, in the context of nurturing plant life indoors. Those caregivers who have comorbidities and physical mobility issues may need to select public gardens that are accessible to wheelchairs.

Other emerging options may include technology-mediated nature immersive experiences to support informal cancer caregivers. Virtual reality natural exposures often masterfully simulate the natural environment [38] and could potentially be utilized to support caregivers who are confined to the home. These experiences offer a connection with the feelings of being in a particular nature environment. The immersive nature of virtual reality, along with its novelty and ease of use, could deliver the benefits of nature and motivate ongoing use [38]. Such technology can be especially appealing to pediatric caregivers since the experience could be shared with the child and discussed [35].

Research is needed that evaluates nature interventions usage for caregiver-cancer patient dyads. Given what is known about the benefits of the natural environment, such research could evaluate important areas such as impact on the relationship and communication, mental health, cognitive effectiveness, quality of life, and potential alleviation or reduction of perceived caregiver burden.

Coincident with shifts in cancer care from the hospital inpatient and outpatient clinic to the home, there is an increased public health impact to ensure that the caregiver’s needs are addressed [10]. Nature is a readily available, easy to access, and cost-effective resource. Investing in studies that evaluate the dose needed, the types of nature that are most effective, that are rigorously designed, and that attend to treatment fidelity are needed.

## 5. Conclusions

Informal home-based caregiving is increasing in prevalence across all racial/ethnic groups, genders, professional work situations, and educational levels [8]. This review of the literature points to the need for more work in the area of cancer caregiver support through exposure to nature interventions. Caregiving is time consuming and physically and emotionally demanding, leaving little time for self-care. Therapeutic natural environments may be a supportive option to restore cognitive and emotional functioning for these caregivers. Nature has long been considered the restorative pause needed from difficult routines, and thus may be taken for granted. Importantly, for family cancer caregivers, the natural environment can vary greatly dependent on the living context and geographic region. For some in urban settings, green space may not be immediately available, whereas for others, the refreshment of nature may be viewed from a window or even virtually. A better understanding of the role the natural environment plays in relieving caregiver burden will help healthcare professionals support friend or family caregivers who provide care to cancer patients. Given the emerging public health issue created by the growth of an aging population with chronic illnesses such as cancer and increasing reliance on aged informal caregivers in the home environment, further research on natural environment supportive therapeutic modalities are recommended.

## Figures and Tables

**Table 1:** Publications addressing nature-relevant interventions for informal family/friend cancer caregivers.

Publications	Sample (Age, Sex, Race, Any Co-Morbid Conditions If Mentioned)	Design (Type of Study)	Type of Intervention	Patient Cancer Diagnosis	Measures Used	Study Outcomes
Carrion-Plaza, et al. (2020). HabitApp: New play technologies in pediatric cancer to improve the psychosocial state of patients and caregivers	39 Spanish pediatric oncology patients and 39 caregivers (61% female, *n* = 24); age range 30–64 years.	Controlled mixed methods pre-post study consisting of 30-minute sessions with pre, during (10 min, 20 min), and post evaluations. Qualitative data were collected during the observation period.	Evaluate use of the HabitApp technological play therapy, a mobile application that permits the observation of animals in their personal habitats from around the world using remote video cameras.	Patients were hematology-oncology patients undergoing cancer treatments—bone marrow transplantation (not at terminal phase).	Observational ad hoc measurement scale (affection, nervousness, proximity to child, reactions, interest, satisfaction) 0–3 ratings; state-trait anxiety inventory; somatic complaints list; positive and Negative experience scale; Mood scale (fear, sadness, happiness, anger); State-Trait Depression Inventory.	Caregivers demonstrated significant improvements in psychosocial factors of affection, proximity, interest, and satisfaction. There were smiling faces and laughter, relaxed conversation, and storytelling between patients and their caregivers.
Lavin, et al. (2020). Determining the effect of group flower arranging sessions on caregiver self-efficacy and stress levels in an in-patient hospice	*n* = 71 family caregivers of patients in patient end of life care. (*n* = 38; 54.3%) were 51 to 76 years old. Majority of the participants (*n* = 65; 92.9%) were females and (*n* = 5, 7.1%) were males. Participants self-identified as Hispanic (*n* = 33, 41.7%), Caucasian (*n* = 22, 31.4%),African American (*n* = 14, 20%), and Asian (*n* = 1, 1.4%).	Mixed methods pre-post design.	Participation in a ‘flowers for healing’ class that taught participants how to arrange flowers. Participants shared their flower arrangement with the patient they cared for.	Terminally ill hospice patients. Type of diagnosis not described although cancer caregiving is alluded to in the literature review.	The revised scale for Caregiving self-efficacy; Likert scale (0–5) used to evaluate stress level, sleep, appetite and eating habits, mood, memory, and sense of wellbeing. Participant satisfaction open-ended comments for future changes and recommendations.	*n* = 55 caregivers did not complete the self-efficacy post-test due to needing to get back to the patient. However, stress levels were significantly decreased in the overall sample. Of those who completed self-efficacy pre- and post-intervention (*n* = 17), there was significant improvement in scores. Qualitative data indicated very positive experience (*n* = 57 reported positive emotions such as relaxation, calming, healing); *n* = 16 identified less worry, reflection; positive sensory experience identified by *n* = 7.
McCullough, t al. (2018). Measuring the effects of an animal-assisted intervention for pediatric oncology patients and their parents: a multisite randomized controlled trial	106 primary parent caregivers (*n* = 60 in intervention group, *n* = 46 in control condition). 92.5% (*n* = 98) primary caregivers were mothers; most of sample ranged between 26–45 years (80%). 67% (*n* = 71) Caucasian; 8% (*n* = 8) African-American; 14% (*n* = 15) Hispanic; 6% (*n* = 6) Other; 6% (*n* = 6) not reported.	Parallel group randomized trial.	Evaluated the effects of an animal-assisted intervention on stress, anxiety, and health-related quality of life in children with cancer and their parents in five U.S. pediatric hospital sites. Intervention occurred in cancer outpatient setting once per week over 4 months approximately depending on cancer treatment schedules. Sessions average 24 min in length.	Children (*n* = 106) were 3–17 years with newly diagnosed cancers (previous 6 weeks); Cancers included lymphomas (*n* = 13, 12%); osteosarcoma (*n* = 6, 6%); Wilms’ tumor 8% (*n* = 8) neuroblastoma (*n* = 2, 2%); sarcomas (*n* = 7, 7%); other (*n* = 15, 14%).	Pediatric Inventory for Parents used to measure stress; 2 items including 4 subscales: communication; emotional functioning, role functioning, medical care. State-Trait Anxiety Inventory (2 20-item scales).	Parents in the intervention group had significant reduction in overall parenting stress post-intervention as compared to the control group (*p* = 0.008). Parents in the intervention group had significant reductions in stressful communications over time (*p* = 0.004), frequency of stressful events related to medical care (*p* = .023), and reduced emotional distress (*p* = 0.002). There were no significant differences in state or trait anxiety pre-post intervention between the groups with increased state anxiety noted in both groups (*p* < 0.001).
Sun, et al. (2020). Barriers and facilitators of adherence to a perioperative physical activity intervention for older adults with cancer and their family caregivers	34 patient-caregiver dyads. Caregivers were 59% female; 82% Caucasian, and 53% were employed.	Qualitative study of the barriers and facilitators to the walking intervention. The caregivers were trained to serve as the patients coach and participated in walking with their patient during the intervention time period.	Evaluation of barriers and facilitators to a physical activity intervention. The peri-operative physical activity intervention was aimed at building the patients physical and psychological function pre-surgery. The dyadic sessions consisted of one-on-one coaching via 5 videoconference and telephone sessions that were delivered pre-surgery, during hospitalization, and 2–4 weeks following surgery.	Lung (*n* = 18; mean age 74 years) and gastrointestinal (*n* = 16; mean age 71 years) cancers.	Data were derived from the physical therapy/occupational therapy notes that were taken during baseline, session 1 before surgery, and inpatient encounter post-surgery.	Family caregivers noted barriers were: co-morbid conditions (pain, arthritis), allergies and sensitivities to walking outdoors, family obligations and work responsibilities, MD appointments, busy schedule, weather, and uneven walking surfaces. Facilitators to outdoor activity included patient wanting to hike in mountains twice weekly; encouragement from patient and benefit such as reduced anxiety.
Turner (2016). The impact of complementary therapies on cancer patient caregivers’ quality of life	Data were evaluated on 120 users of therapies and 120 non-user cancer caregivers from a large cancer center. Complementary therapy users were primarily female (68%), Caucasian (82%), college educated (64%), and living with a partner (71%).	Secondary analysis of cross-sectional data that compared quality of life data between cancer caregivers who did vs. those who did not participate in cancer center complementary therapies program.	Complementary therapy classes offered to the cancer patients and their caregivers were divided into healing (art, gardening, movement, music, photography, and writing) and fitness (aquatic therapy, cycling, pilates, strength and fitness, walking, and yoga).	Cancer patients at large cancer center.	51-item quality of life survey (36 quality of life questions; 15 demographic items) with a single open-ended question about the impact of complementary therapies. User data from records about complementary therapy usage.	Complementary therapy uses reported significantly better scores on relationship with others, mental functioning, emotional state, and attitude toward life as compared to non-complementary therapy users. 40% of the use (highest reported along with arts program) were hope blooms gardening. 20% of users also participated in walking and cycling.

## Supplementary Materials

The following are available online at https://www.mdpi.com/article/10.3390/ijerph182111124/s1, Table S1: Full keyword search terms for each database.
