# Supplementary material for: An Evaluation of Natural Environment Interventions for Informal Cancer Caregivers in the Community
Source: Int J Environ Res Public Health. 2021 Oct 22;18(21):11124. doi: 10.3390/ijerph182111124 (PMC8583496; doi:10.3390/ijerph182111124)
Supplement: Supplementary file 1 [file ijerph-18-11124-s001.zip › ijerph-1436756-supplementary.pdf]

Table S1. Full keyword search terms for each database.

Database	Terms
PubMed	((((Caregiv* OR "Caregivers"[Mesh]) AND (family OR "Family"[Mesh] OR friend "Friends"[Mesh] OR spous* OR “significant other” OR "Spouses"[Mesh] OR partner OR husband OR wife OR dyad OR relative OR informal OR unpaid OR lay)) AND (Cancer* OR "Neoplasms"[Mesh] OR oncolog* OR neoplasm*)) AND (Nature OR park* OR greenspace* OR “green space” OR “green spaces” OR tree* OR garden* OR landscape* OR “nature sound” OR “nature sounds” OR "Nature"[Mesh] OR "Parks, Recreational"[Mesh] OR "Trees"[Mesh] OR "Gardens"[Mesh] OR “natural environment” OR horticulture OR outdoor* OR environment OR outside OR ecolog* OR "Ecology"[Mesh] OR ocean OR "Oceans and Seas"[Mesh] OR lake OR "Lakes"[Mesh] OR river OR "Rivers"[Mesh] OR forest* OR "Forests"[Mesh] OR wood* OR "Horticulture"[Mesh] OR "Environment"[Mesh] OR neighborhood OR sidewalk* OR “botanical garden” OR “botanical gardens” OR flower OR floral OR plant*)) AND (Therap* OR "Therapeutics"[Mesh] OR "Recreation Therapy"[Mesh] OR “recreation therapy” OR “horticulture therapy” OR “Horticultural Therapy"[Mesh] OR "Complementary Therapies"[Mesh] OR “complementary therapies” OR “alternative therapies” OR “quality of life” OR QoL OR "Quality of Life"[Mesh] OR integrative OR “integrative therapies” OR “integrative therapy”)
CINAHL	(caregiv* OR (MH "Caregivers")) AND (family OR (MH "Family") OR friend OR spous* OR (MH "Spouses") OR “significant other” OR (MH "Significant Other") OR partner OR husband OR wife OR dyad OR relative OR informal OR unpaid OR lay) AND (cancer* OR neoplasm* OR oncolog* OR (MH "Oncology") OR (MH "Neoplasms")) AND (nature OR landscape* OR tree* OR park* OR greenspace* OR “green space*” OR garden* OR “nature sound*” OR “natural environment” OR (MH "Natural Environment") OR horticulture OR outdoor* OR environment* OR outside* OR outdoor* OR ecolog* OR ocean* OR lake* OR river* OR forest* OR wood* OR neighborhood OR “botanical garden” OR flower* OR plant OR (MH "Flowers") OR (MH "Plants")) AND (therap* OR “horticulture therap*” OR “recreation therap” OR “complementary therap*” OR “alternative therap*” OR “quality of life” OR QoL OR “integrative therap*” OR (MH "Quality of Life") OR (MH "Alternative Therapies") OR integrative)
AltHealthWatch	caregiv* AND (family OR friend OR spous* OR “significant other” OR partner OR husband OR wife OR dyad OR relative OR informal OR unpaid OR lay) AND (cancer* OR neoplasm* OR oncolog*) AND (nature OR landscape* OR tree* OR park* OR greenspace OR “green space*” OR garden* OR “nature sound*” OR “natural environment” OR ocean* OR lake* OR river* OR forest* OR wood* OR outdoor* OR outside* OR ecology* OR neighborhood OR “botanical garden” OR flower OR floral OR plant*) AND (therap* OR “horticulture therap*” OR “recreation therap” OR “complementary therap*” OR “alternative therap*” OR “quality of life” OR QoL OR “integrative therap*” OR integrative)
Cochrane	caregiv* AND (family OR friend OR spous* OR “significant other” OR partner OR husband OR wife OR dyad OR relative OR informal OR unpaid OR lay) AND (cancer* OR neoplasm* OR oncolog*) AND (nature OR landscape* OR tree* OR park* OR greenspace OR “green space*” OR garden* OR “nature sound*” OR “natural environment” OR ocean* OR lake* OR river* OR forest* OR wood* OR outdoor* OR outside* OR ecology* OR neighborhood OR “botanical garden” OR flower OR floral OR plant*) AND (therap* OR

Database	Terms
	“horticulture therap*” OR “recreation therap” OR “complementary therap*” OR “alternative therap*” OR “quality of life” OR QoL OR “integrative therap*” OR integrative)
Scopus	caregiv* AND (family OR friend OR spous* OR “significant other” OR partner OR husband OR wife OR dyad OR relative OR informal OR unpaid OR lay) AND (cancer* OR neoplasm* OR oncolog*) AND (nature OR landscape* OR tree* OR park* OR greenspace OR “green space*” OR garden* OR “nature sound*” OR “natural environment” OR ocean* OR lake* OR river* OR forest* OR wood* OR outdoor* OR outside* OR ecology* OR neighborhood OR “botanical garden” OR flower OR floral OR plant*) AND (therap* OR “horticulture therap*” OR “recreation therap” OR “complementary therap*” OR “alternative therap*” OR “quality of life” OR QoL OR “integrative therap*” OR integrative)
